# An innovative nurse practitioner-led service for children from families living in housing instability

**DOI:** 10.1017/S1463423625000118

**Published:** 2025-02-27

**Authors:** Alicia Bell, Yvonne K. Parry, Matthew Ankers, Nina Sivertsen, Eileen Willis, Sally Kendall, Huahua Yin

**Affiliations:** 1 College of Nursing and Health Sciences, Flinders University of South Australia, GPO Box 2100, Adelaide 5001, Australia; 2 Caring Futures Institute, Flinders University of South Australia, GPO Box 2100, Adelaide 5001, Australia; 3 UiT Arctic University of Norway, Faculty of Health Sciences, Hammerfest, Kvaløya, Finnmark, Norway; 4 Central Queensland University; 5 University of Kent, Canterbury, Kent CT2 7NZ, United Kingdom

**Keywords:** access, children, children’s health, housing instability, nurse practitioner, primary care

## Abstract

**Aim:**

To report on the design and results of an innovative nurse practitioner (NP)-led specialist primary care service for children facing housing instability.

**Background:**

During 2017–2018, children aged 0–14 years represented 23% of the total population receiving support from specialist homeless services in Australia. The impact of housing instability on Australian children is considerable, resulting in disengagement from social institutions including health and education, and poorer physical and mental health outcomes across the lifespan. Current services fail to adequately address health and educational needs of children facing housing insecurity. Research identifies similar circumstances for children in other high-income countries. This paper outlines the design, and reports on results of, an innovative NP-led primary care service for children facing housing instability introduced into three not-for-profit faith-based services in one Australian state.

**Methods:**

Between 2019 and 2021, 66 children of parents experiencing housing instability received standardized health assessment and referral where appropriate by a NP. Data from the standardized tool, such as condition and severity, were recorded to determine common conditions. In addition, comprehensive case notes recorded by the NP were used to understand potential causes of conditions, and referral needs, including potential barriers.

**Findings:**

The 66 children assessed were aged between 7 weeks to 16 years. Developmental delay, low immunization rates, and dental caries were the most common conditions identified. Access to appropriate services was inhibited by cost, disengagement, and COVID-19.

**Conclusion:**

Given their advanced skills and knowledge, embedding NPs in specialist homeless services is advantageous to help vulnerable children.

## Introduction

On census night in 2016, Australia recorded 19 400 children, aged 0–14 years, as homeless; this figure represented 17% of the total homeless population (Australian Institute of Health and Welfare [AIHW], 2020). In 2017–2018, children in the same age range represented 23% or 65 600 of the total population receiving help from specialist homelessness services, of which 45% or 29 600 did so for interpersonal reasons related to domestic and family violence or family breakdown (AIHW, 2020). A significant predictor of homelessness is housing stress, which is described as an adult(s) spending 30% or more of their income on accommodation (AIHW, 2020). Housing stress can cause financial strain and conflict and impact a family’s ability to buy food and clothes or pay for utilities (AIHW, 2020). Housing stress affected one in five Australian children in 2016 (AIHW, 2020). The more recent impact of COVID-19 has only exacerbated several of these factors including the cost of accommodation, access to employment, and the risk of domestic violence. This suggests that post-pandemic figures regarding housing stress, homelessness, and their wider impacts will be worse for families including their children (Boyrs, 2020; House of Representatives Standing Committee on Social Policy and Legal Affairs, 2020; Thornton *et al*., 2020; Henriques-Gomes, 2021).

Australian children accessing specialist homeless services (normally as part of a disadvantaged family) often have unmet health and educational needs (Parry *et al*., 2020b; AIHW, 2020). For example, housing instability can result in poorer academic outcomes, due to moving frequently between accommodations or schools (high mobility), poor school attendance, disturbed sleep, and/or lack of space to study. The cost associated with transport, school supplies, and uniforms can also reduce a child’s ability to engage with peers and education (AIHW, 2020; The Smith Family, 2021). Similarly, health care may be delayed or irregular due to the cost and/or ability to access services, which, in Australia, can result in disjointed care from emergency departments, which is provided free of charge, in comparison to general practitioners (GPs) who may charge a fee (Parry *et al*., 2015; Parrott, 2020). Children’s disengagement from social institutions such as health and education, when coupled with housing instability, can result in poorer physical and mental health outcomes, as well as missed developmental milestones, that together lead to poorer outcomes across the lifespan (Voices of Youth (VoY), 2019; AIHW, 2020; Parrott, 2020; Thornton *et al*., 2020; The Smith Family, 2021). Essentially, current services are poorly designed to address the needs of children living in housing instability and targeted primary health care is paramount to prevent cumulative harm to underserved children (Parry *et al*., 2020b; Thornton *et al*., 2020; Molokhia and Harding, 2021).

The majority of current services for homeless families and individuals in Australia are outsourced to private providers (Pawson *et al*., 2018). A number of these services are faith-based not-for-profit organizations that either operate alone or are loosely networked with other organizations. Their activities are governed and constrained by the terms of their grants, won through tenders from either the State or the federal government, usually on a three- or five-year basis, making long-term planning difficult (Pawson *et al*., 2018). These services provide a central meeting place for vulnerable populations, including families with children and women escaping domestic violence, where a range of supports are provided from legal, social, financial, and accommodation assistance (Pawson *et al*., 2018).

In South Australia, child health and development checks are predominantly completed by Child and Family Health (CaFH) nurses and GPs in the child’s first 24 months. These checks are routine and occur either in the home or within CaFH centres (Cahill *et al*., 2022). Research indicates varied success in reaching this population for a variety of reasons, such as poor engagement, particularly within vulnerable communities (Moreno-Betancur *et al*., 2023). Specific to this study, families experiencing housing instability often miss scheduled appointments or may have disengaged with primary healthcare providers, and rarely access early childhood education, therefore necessitating comprehensive health and developmental assessment. This paper reports on the pilot nurse practitioner (NP)-led service, which was embedded in a homelessness service, and illustrates the outcome of the NP clinic.

## Methods

The methods for this study involved establishing the NP-led clinic, collection and analysis of data, and reporting. These are outlined below.

### The establishment of the NP-led primary health child clinic

Given the potential for NP to provide comprehensive primary care to vulnerable groups, we embedded an NP service, within a specialist faith-based homeless service, for children from families suffering housing instability in one Australian state. Funding for the service came from Medicare rebates that NPs received for the care they provided and from three research grants that evaluated the service. Once the NP-led clinic was established, case managers from the homeless service introduced the clinic to all families presenting to the service, who had children in their care, and invited them to seek voluntary primary care for their children from the NP service. Consenting families had an appointment with the NP coordinated by their case manager. The NP initially met the family with the case manager to help build rapport. Following this, the NP performed a comprehensive assessment of the child and, if required, would call local service providers to find appropriate referral services and enquire about wait times and associated costs. Additionally, some families required home visits by the NP as they lacked access to transport or public transport was not efficient. This was especially so during COVID-19. In cases where the NP made referral appointments to allied health or medical specialists, the NP or case managers from the homeless service often drove families to appointments.

An NP experienced in assessing health, development, and behaviour, with the ability to refer to specialists as required, is a logical innovation to service this population. Specifically, NPs are registered nurses with additional master’s level degrees and specialized education and experience, who work in an advanced practice capacity across a variety of settings (Hollinghurst *et al*., 2006; Jennings *et al*., 2015; Kilpatrick *et al*., 2021). The nurse practitioner’s advanced scope of practice ensures they can perform comprehensive assessment, order investigations, diagnose, and prescribe medications, within a defined area of expertise (Dwyer *et al*., 2021; Harvey *et al*., 2021).

NPs have been identified as professionals who provide high-quality care and significant improvement in access to health care, particularly for people from disadvantaged backgrounds, marginalized populations, Aboriginal and/or Torres Strait Islander groups, and culturally diverse groups (Barrett *et al*., 2015; Australian Primary Health Care Nurse Association (APNA), 2019; Dwyer *et al*., 2021; Harvey *et al*., 2021). Nurse practitioners are also foundational in providing professional education to patients (clients/consumers) and interprofessional teams. They can assist in the expansion of clinical expertise within interdisciplinary teams through mentoring and sharing advanced clinical knowledge (Perfetto *et al*., 2018). The care provided by an NP is also cost effective for the healthcare system. For example, a comprehensive health assessment of at least 40 min performed by an NP in Australia has a Medicare rebate of $73.80, with a similar 40-minute assessment from a GP costing the taxpayer via Medicare, $122.15 (Department of Health and Aged Care, 2024b). The rebate offered by Medicare to the NP for services rendered is approximately half that of a GP, who also regularly charges a gap fee.

### Data collection

In total, 66 children referred by the specialist homeless service were reviewed by the NP (some also having multiple follow-up sessions) between December 2019 and March 2021. The nurse practitioners collected multiple sources of data throughout their assessment and on follow-up, to build a comprehensive picture of clients, and their needs. Data collection included the use of a standardized child assessment tool developed by the Australian Government’s Department of Health, titled ‘Medicare Health Assessments for Aboriginal and Torres Strait Islander People’ (Department of Health and Aged Care, 2024). This standardized assessment tool incorporated a well-balanced overview of a child’s health while also focusing on culture and potential Indigenous health needs as many clients accessing the NP service identified as Aboriginal and/or Torres Strait Islander families. Additionally, the NPs recorded comprehensive case notes about the child’s appearance, behaviour, and interactions from observations and discussions with them and/or their parents. The NP also asked parents about their current housing status, guardianship, and recent use of medical services. Data from the standardized health assessment, such as identified conditions and their severity, were recorded manually into a Microsoft Excel spreadsheet. The severity of an identified condition was established using a scale of 1 to 3, where:

- 1 represented a condition that required treatment by the NP and a general medical practitioner (GP) or referral to a specialist but was non-urgent.
- 2 represented a condition that required treatment by the NP, GP, or similar or referral to a specialist in the near term (as soon as possible without being urgent).
- 3 represented a condition that required treatment by the NP, GP, or similar or referral to a specialist or emergency department immediately.

Health assessment data were cross-referenced with case notes and field observations to build a more comprehensive representation of the client’s common conditions, referral needs, and social situation. The use of multiple data points to explore children, who accessed a specialist homeless service, meant that these findings could be triangulated, which helped confirm reported outcomes and increased the research validity (Patton, 2015; Yin, 2014). These data were supplemented by information collected by the homeless service providers such as the date of the visit and housing situation.

### Data analysis

Data were analysed using IBM SPSS Statistics version 28.0.0.1(Armonk, NY: IBM Corp.). Descriptive statistics were conducted. Continuous data were presented as mean and standard deviation (SD), and categorical data were presented as percentages and frequencies.

## Results

### Participants

The average age of these 66 children was 6.8 years (SD = 4.4) with a range from 7 weeks to 16 years. The majority (60.0%, n = 36 from 60 responses) lived in emergency motel accommodation, and most of them resided with their mother (84.6%, n = 55 from 65 responses). Among the 66 children, 25 were recorded as being exposed to domestic violence, and 16 were identified as Aboriginal or Torres Strait Islander families (24.2%). Table 1 summarizes these demographic variables.

**Table 1.** Demographics of participants

Variables	n	%
Current housing situation (n = 60)^*^		
Emergency motel accommodation/emergency housing	42	70.0
Current housing crisis/live in squalor	3	5.0
Temporary housing/supported temporary housing	3	5.0
Supported housing	10	16.7
Supported private rental	2	3.3
Current carer of child (n = 65)^*^		
Mother and father	3	4.6
Mother and partner	1	1.5
Mother and stepfather	2	3.1
Mother	55	84.6
Father	1	1.5
Grandmother	3	4.6
Child exposed to domestic violence (n = 32)^*^		
Yes	25	78.1
No	7	21.9
Aboriginal or Torres Strait Islander (n = 66)		
Yes	16	24.2
No	49	74.2
Unclear	1	1.5

* *Note*: Data are incomplete in some instances, and missing values were not included; valid percent was reported.

#### Children’s immunization status and health conditions

Most children (81.5%, n = 53) did not have a documented up-to-date immunization record. Table 2 showed that 64.6% (n = 42) of children were assessed as having a physical or mental health condition/conditions, and among these, 81% (n = 34) of children required intervention, while 32.3% (n = 11) needed an urgent referral to a health professional. The common health conditions identified included: dental (n = 14), developmental delay (n = 6), ear, nose, and throat (ENT) (n = 6), and mental health (n = 6).

**Table 2.** Children’s immunization status and health conditions

Variables	n	%
Immunization status up-to-date as confirmed by sighting official documentation (n = 65)		
Yes	12	18.5
No	53	81.5
Health conditions identified by NP (n = 65)		
Yes	42	64.6
No	23	35.4
Leading health conditions identified by NP (n = 42)		
Dental	14	33.3
Developmental delay/gross motor delay	6	14.3
ENT (ear infection, foreign body, enlarged tonsil)	6	14.3
Mental health	6	14.3
Severity of health conditions identified^*,†,‡^ (n = 34)		
Minor	12	35.3
Moderate	11	32.3
Severe	11	32.3

*Note*: Missing values were not included; valid percent was reported.

Minor: a condition that is non-urgent and required treatment by NP or review by GP and does not need an immediate referral or referral response. Therefore, the child can wait on the public health waiting list.

Moderate: represented conditions that required treatment by the NP or review by a GP, allied health, or specialist in the near term (as soon as possible without being urgent), for example, low-level eczema, and chest infection.

Severe: represented a condition that required treatment by the NP or review by a GP, allied health, or specialist immediately, for example, an x-ray for a broken foot (previously untreated) or enlarged tonsils.

* If the health condition identified did not need any referrals, it was not considered as any level of severity. Level of severity.

† A child could be identified with more than one condition; if so, the highest level of severity was recorded for this child.

‡ Severity of conditions.

**Table 3.** Service provided by nurse practitioner

Variables	n	%
Home visit (n = 65)		
Yes	49	75.4
No	16	24.6
If Yes, reasons for home visit^*^ (n = 47)		
No transport	18	38.3
COVID-19 pandemic	17	36.2
Other (various complex reasons, e.g. domestic violence, mental health problems, anxiety, not comfortable to visit the service)	17	36.2
Treatment provided by NP (n = 66)		
General health assessment^†,‡^	47	71.2
General physical assessment^†^	15	22.7
No assessment^§^	4	6.1
Referral arranged by NP (n = 66)		
Yes – Direct referral generated and sent	27	40.9
No – Referral recommended (advice offered to parents, but consent for referral not provided)	7	10.6
No – Referral not needed	31	47.0
Other (Already had active referral)	1	1.5

*Note*: Missing values were not included; a valid percent was reported.

* A child may have more than one reason; two children who required home visits did not provide any reasons.

† General health assessment/general physical assessment may include discussion (e.g. discussion about treatment plan), reassurance, etc.

‡ One child had a partial general health assessment due to not being very receptive.

§ No assessment included one child who was well connected appropriate with services; thus, no assessment was needed; one child was unable to do an assessment; one child had a tele-health appointment.

#### Service provided by nurse practitioner

The majority of children required assessment by the NP in their home or residence (75.4%, n = 49), the common reasons for home visits were no access to transport (38.3%, n = 18) or the restrictions caused by the COVID-19 pandemic (36.2%, n = 17). Approximately, 93.9% (n = 62) of children received general health/physical assessment, and 40.9% (n = 27) were provided with referrals. Table 3 summarises the services provided by the NP.

## Discussion

### Health conditions

#### Common health conditions of children living in housing instability

This study showed that low immunization rates, dental conditions, ENT conditions, developmental delay, and mental health were among the most common issues identified in children who accessed the NP through the specialist homeless service. These conditions are supported by National and International research in the field (Sheau-Huey and DiMarco, 2009; Parry *et al*., 2020a; 2020b). They are discussed below in detail.

#### Immunization

Over 80% of children seen by the NP at the specialist homeless service did not have appropriate documentation for their child’s immunization status. Furthermore, unimmunized children required a full immunization catch-up plan in line with the ‘catch-up schedule’ as outlined in the National Immunization Program (Department of Health and Aged Care, 2023). The cause of children missing scheduled immunizations was often related to poor health education, poor access to transport, cost (GPs charging a gap fee, for example), and/or the transient nature of this group’s housing. It can also be difficult for children and families living in housing instability to coordinate appointments for vaccinations, as they can require an appointment with a GP, then access to a pharmacy, and finally access to someone who can administer the dose. This process was made significantly more difficult during COVID-19, due to the closure of many services. Unfortunately, vaccination administration by the NP could not be offered at the time, given the capacity of the service, the logistics of storing vaccines, refrigeration monitoring, and the risks involved with storing needles and syringes on site. If the service is expanded in the future, vaccination will be high on the agenda to benefit the children and clients utilizing the service.

#### Dental health

Poor oral health among those living in housing instability was another common concern, which is consistent with the previous study (DiMarco *et al*., 2010). This was due to many children living in housing instability having poor dental hygiene practices, sub-optimal diets, and poor access to care (DiMarco *et al*., 2009). As a result, referral for dental intervention was a common practice for the NP. Initially, with no direct referral process, the NP was able to advocate for dental assessment through the South Australian School Dental Service (SASDS), to which phone numbers were provided to families to make appointments for free dental assessment and treatment. However, compliance was low throughout the first eight months of the NP service, which led to the development of a direct referral process through the specialist homeless service to ensure the children were seen by SASDS as a high priority, which improved appointment attendance.

#### Ear, nose, and throat

ENT conditions are common in children and can result in high morbidity when undetected (Hayois and Dunsmore, 2023), highlighting the importance of assessment by appropriately trained paediatric NPs for this vulnerable population. A total of 14.3% of children were identified as having an ENT condition. These conditions included acute otitis media, obstructive sleep apnoea, foreign body in the ear canal, and tonsil hypertrophy. All ENT conditions were identified as a direct result of the holistic NP assessment and may have remained unidentified, without this service.

With the skills and knowledge to assess a paediatric ear, identify and manage potential problematic conditions, consider diagnostic differentials, and provide antibiotic coverage when required, NPs are well situated in this unique primary health setting to minimize unnecessary ENT complications. Undiagnosed ear infections can result in hearing loss, contribute to disruptive behaviour in the classroom, and potentially impact school performance (Hayois and Dunsmore, 2023), highlighting the critical need for appropriate primary care for these children.

#### Developmental delay

Children living in housing instability are at significantly greater risk of developmental delay than children from higher socio-economic backgrounds (Ginn *et al*., 2020). This trend was also observed in children attending the NP clinic in this study, with 14.3% of children identified as having health conditions leading to developmental delay. In Australia, the process of gaining a formal diagnosis of autism spectrum disorder (as one example) requires an assessment by two separate, accredited clinicians such as a paediatrician, psychologist, psychiatrist, or speech pathologist (Connect Allied Health, 2020). However, the process of private referral and assessment is expensive, with an average cost to Australian families of $2000, and initial recognition and referral are often delayed meaning children can miss out on timely and appropriate health care (Taylor *et al*., 2016; Connect Allied Health, 2020; Parry *et al*., 2020a; Autism SA, 2024). This is especially true for children of families in housing instability as financial disadvantage further restricts access to these services, despite the cohort’s higher identified need (Parry *et al*., 2016).

Referral to the public health sector for paediatrician review is also problematic, as the current wait time for such appointments can be in excess of 18 months (South Australian Health, 2020). However, many of the families who took part in our study lived in temporary emergency accommodation and had often relocated by the time triaging documentation for specialist appointments was mailed out, or families had moved outside of the hospital catchment area to which they had originally been referred, resulting in cancellation of the referral (Government of South Australia, 2024). Hence, the transient nature of existence for some families suffering from housing instability exacerbated disengagement from the health system. An additional issue of note is the high cost of private-sector care, which places diagnosis through this method, out of reach for many. Despite this, official diagnosis is crucial, as it grants families access to financial support for treatment and cost-effective therapy, such as the Australian National Disability Insurance Scheme (NDIS) (NDIS, 2024). It is well researched that early intervention for developmental delay is imperative to improve outcomes for children (Lipkin *et al*., 2020). A delay in diagnosis and therapy means many families increasingly struggle to cope, while the impact on the child’s longer-term development and outcomes is negative, pointing to a significant gap in health equity for people from lower socio-economic positions.

#### Mental health

Consistent with our study, previous research found that children living in housing instability are at high risk of developing mental health and behavioural conditions, such as depression, anxiety, and ADHD (DeSocio and Hootman, 2004; Agorastos *et al*., 2019), which can affect them long term and into adulthood. Trauma-based responses to housing instability and the stressors leading to homelessness can have a profound effect on children, manifesting in a range of adverse behaviours or delay in development (Agorastos *et al*., 2019). It is critical that children displaying emotional distress or associated behaviours receive timely access to psychological support.

#### Health access issues

This study showed most families requested a home visit for the NP service for various reasons, demonstrating the difficulties they encounter accessing health services. This result further supports the previous research indicating that major barriers to primary healthcare services for children and their families living in housing instability were poor engagement and lack of access (Davies and Wood, 2018, Parry *et al*., 2020b). The reasons for these barriers are complex and often multifaceted and can include personal exposure to significant and ongoing trauma such as domestic violence as well as mental health and/or substance abuse issues, and financial stress (DiMarco *et al*., 2010). Families can also feel stigmatized by health professionals due to their living arrangements and/or personal issues. As a result, parents living in housing instability are reluctant to seek assistance when required due to fear of being judged or having their children removed from their care (Davies and Wood, 2018). An additional issue is the overburdened public health system that results in long wait times to see specialists, which also impacts people’s ability to access affordable health professionals.

A number of children seen by the NP service required rapid intervention but lacked sufficient resources to be reviewed privately by a GP or specialist despite presenting with symptoms too severe to wait for review within the public sector. In these situations, the NP used advanced practice expertise and knowledge of the local healthcare system to help navigate and fast-track assessment and referral to specialist services. Specifically, this process involved contacting specialists directly and discussing individual scenarios, which expedited appointments for vulnerable children who would otherwise be negatively impacted with potential long-term implications if they had used the designated pathway. This type of innovative care is required in the homelessness setting to ensure children who have previously slipped through healthcare gaps are captured and their health needs addressed. It also identifies how NPs, with their advanced scope to diagnose and treat, their ability to discuss clients with specialists on an in-depth level, and their knowledge of local health systems, ensure that clients get appropriately linked to services.

### Nurse practitioners are well placed to help underserved populations

A key element to the success of the NP-led service in the homelessness sector is their knowledge of the local public health system. The number of children presenting to the service with previously undiagnosed or inadequately managed chronic conditions was significant. Therefore, embedding an NP in this environment, who has advanced nursing and assessment skills as well as knowledge and experience in the local public health sector, resulted in appropriate referral and access to public health services, at no financial cost to the patients. The NP was also able to provide continuity of care and become a support person for families to liaise with, ensuring appointments were attended. It is well established that continuity of care is a significant component of quality health care (Davis *et al*., 2021).

Referral compliance is also a common barrier to care as families living in housing instability and poverty often do not have appropriate access to transport (Mallett *et al*., 2011; AIHW, 2020; Nolan-Isles *et al*., 2021; Sivertsen *et al*., 2022). Missed appointments are a persistent problem across healthcare settings, and result in negative outcomes for providers and patients (Crable *et al*., 2021). The issue of referral compliance in this research included client non-attendance or last-minute cancelations. A major cause of this was a lack of transport impacting people’s ability to attend health appointments. This was overcome by the NP through a collaborative and team-based approach with the specialist homeless service who provided transport to the appointment. This improved the health outcomes for the children who accessed the services and supported the parents, many of whom were struggling to obtain the appropriate health services needed to care for their children. This is consistent with findings from research identifying that access to transportation options, interagency communication and coordination, continuity and consistency of care, one-on-one case management, and respect for and understanding of factors competing with healthcare access enable access and enhance the experience and trust of Aboriginal people in health services (Nolan-Isles *et al*., 2021).

A final issue for this cohort concerning access was that many families living in homelessness and housing instability do not have or could not provide their Medicare details, which may result in the clinician denying service, or being unable to access Medicare rebates for services rendered. Given the value of the NP services, there is potential for the Australian Government going forward to review Medicare rebates and the way in which they are allocated, so that professionals such as NP can better cover their expenditures when providing care for difficult-to-serve populations. This proactive step would help to circumvent the poor health outcomes for children living in housing instability and enhance the ability of NPs to facilitate care to this cohort. Consequently, due to the current MBS rebates, a fee-for-service would need to be charged to ensure the service offered is financially sustainable for the clinician (Australian Primary Health Care Nurse Association (APNA), 2019).

### A final note – extending NP to provide primary care to vulnerable populations

This initiative of embedding a paediatric-trained NP into a homeless service to provide clinical care to children of parents living in housing instability demonstrates the value of extending funding through Australia’s healthcare system to cover primary and extended care for this population. As noted, this population often lacks access to a family GP, particularly in cases of domestic violence or because of the increased costs of GP visits. Given this population often resorts to presenting at the accident and emergency department for their primary care, the establishment of clinics linked to welfare service provisions enables a more holistic service. With the introduction of tele-health MBS item numbers during the COVID-19 pandemic, NPs can help clients by assessing and managing acute and chronic health conditions and, as a result, prevent unnecessary emergency department presentations. This service also addresses the current healthcare shortage of GPs and the increasing costs of primary care for families (Department of Health and Aged Care, 2024a).

### Conclusion

This research examined an NP service that addressed a gap in current health service delivery for children experiencing vulnerability, living in housing instability that, if left unattended, has the potential to cause life-long health issues. Without NPs, these children remain invisible to the health system with unaddressed health and developmental needs. NPs are perfectly positioned within the primary healthcare setting to help care for these underserviced populations. Helping vulnerable families to utilize the NPs’ advanced clinical assessment skills and expanded knowledge of local primary health networks, has both increased access to health services in the homelessness sector as well as being extremely rewarding for the clients and professionals involved. Going forward, developing professional relationships with members of various disciplines within the primary healthcare sector and learning about appropriate services available to help low socio-economic families access basic healthcare services is vital.

### Limitations

The study drew on clients from one specialist homeless service, which had a defined catchment area within the metropolitan area of one Australian state. While findings might be transferrable to other states and jurisdictions, it is difficult to generalize to larger cohorts given the single setting and small cohort. Despite this, we believe our study offers invaluable insight into health access for a vulnerable group, who are clearly in need.
